# Adaptation to Climate Change in Viticulture: The Role of Varietal Selection—A Review

**DOI:** 10.3390/plants14010104

**Published:** 2025-01-02

**Authors:** Miguel Baltazar, Isaura Castro, Berta Gonçalves

**Affiliations:** 1 Centre for the Research and Technology of Agro-Environmental and Biological Sciences (CITAB), University of Trás-os-Montes e Alto Douro (UTAD), 5000-801 Vila Real, Portugal; icastro@utad.pt (I.C.); bertag@utad.pt (B.G.); 2 Institute for Innovation, Capacity Building and Sustainability of Agri-Food Production (Inov4Agro), University of Trás-os-Montes e Alto Douro (UTAD), 5000-801 Vila Real, Portugal; 3 Department of Genetics and Biotechnology, University of Trás-os-Montes e Alto Douro (UTAD), 5000-801 Vila Real, Portugal; 4 Department of Biology and Environment, University of Trás-os-Montes e Alto Douro (UTAD), 5000-801 Vila Real, Portugal

**Keywords:** grapevine, climate change, abiotic stress, mitigation strategies, varietal selection, molecular mechanisms

## Abstract

Viticulture faces unprecedented challenges due to the rapidly changing climate, particularly in regions like the Mediterranean Basin. Consequently, climate change adaptation strategies are crucial in viticulture, with short-term strategies being widely used despite increasing concerns about their sustainability, and long-term strategies considered promising, though costly. A promising but understudied strategy is varietal selection, as grapevines exhibit vast intervarietal diversity with untapped potential for climate-resilient varieties. By integrating research across plant physiology, biochemistry, histology, and genetics, we can better understand the traits behind the grapevine’s capability for adaptation. Several traits, including morphological, physiological, and molecular aspects, have been shown to be crucial in adapting to environmental stresses such as drought and heat. By studying the abundant grapevine intervarietal diversity, the potential for viticulture adaptation to climate change through varietal selection is immense. This review article focuses on the potential of varietal selection in the adaptation of viticulture to climate change. For this, we will delve into the research regarding how climate affects grapevine growth and grape quality and how the grapevine responds to stress conditions, followed by a summary of different climate change adaptation strategies of viticulture. Finally, we will focus on varietal selection, discussing and summarizing different studies surrounding grapevine variety behaviour.

## 1. Introduction

The grapevine (*Vitis vinifera* L.) is one of the most important fruit crops worldwide [1] and viticulture is a major socioeconomical activity in most parts of the world [2]. The total vineyard area worldwide has been estimated at 7.2 million hectares, and by 2023 the wine production was presumed to be around 237 million hectolitres [3,4], with the European continent being the major contributor due to renowned wine-making countries, such as Spain, France and Italy, leading the production charts [4]. Although these regions present diverse climatic characteristics, they belong to the Mediterranean Basin and are considered of Mediterranean climate, with warm dry summers and wet winter periods [5,6]. Grapevines grown under these conditions often face numerous environmental constraints, which typically increase the quality of grapes, and, in turn, enrich the quality of the produced wines [7]. Nevertheless, the typical climatic conditions of the Mediterranean Basin are foreseen to alter significantly due to climate change, with projections identifying this region as a prominent “hot spot” [8,9]. Mediterranean countries are expected to experience substantial temperature rises, extended periods of severe drought, increased levels of ultraviolet (UV) radiation, and a higher occurrence of extreme weather events [8,9,10,11,12]. As such, research regarding climate change and viticulture has been one of the hottest topics in the past years, focusing on understanding how climatic conditions modulate and affect the grapevine and grape quality, while also developing mitigation strategies to help reduce the projected negative effects [13,14,15,16]. One of the mitigation strategies which is consistently mentioned in recent research as a promising tool for viticulture against climate change is varietal selection [17,18,19,20]. Research surrounding this topic is usually focused on phenological data, which, despite being extremely important in the context of climate change, discounts other aspects of plant behaviour with potential for adaptation [21]. Therefore, the objective of this review is to provide further insight on varietal selection as a climate change mitigation strategy for viticulture. For this, an extensive search was conducted for relevant publications, focusing on (i) climate change impacts on viticulture and adaptation strategies; (ii) the effects of abiotic stress on grapevine growth, development and grape quality; and (iii) comparative studies on the behaviour of different grapevine varieties under the same conditions. To better understand the capability of this strategy, information was compiled according to the following topics: how climatic conditions modulate the growth and development of the grapevine and the quality of the grape; the mechanisms underlying grapevine response to stress; the different adaptation strategies of viticulture to climate change, with a special focus on the potential of varietal selection; and lastly, research undertaken in understanding the behaviour of different grapevine varieties in the same conditions, with the last part focusing on the potential molecular mechanisms underlying it.

## 2. Effects of Climate Change on Viticulture—An Overview

Viticulture is highly dependent on environmental variables, as the growth and development of the grapevine is influenced by a complex interactive system of climate, soil, geography, variety and cultural practices known as terroir [22,23,24]. This interactive system is recognized in all viticultural regions worldwide, especially in European countries where tradition is also a determinant in the quality of the produced wines [25]. Despite the grapevine’s adaptability to different environments, their growth conditions have a great impact on the production of wine [13,24,25,26,27]. This is one of the reasons why researchers have been focusing on understanding how the climatic components of the *terroir* affect the growth and development of the grapevine and grape quality, as well as how the predicted changes in climate will affect viticulture as a whole [8,28,29,30,31].

As stated by the Intergovernmental Panel on Climate Change (IPCC), climate is predicted to shift due to anthropogenic effects, with temperature expected to keep rising [32]. In fact, higher temperatures and longer drought periods are some of the worse of the anticipated climate change consequences, and are expected to severely impact several agronomic sectors worldwide, especially those in the Mediterranean Basin [9,32,33], where harsher conditions and the occurrence of extreme events have been documented in recent years [34,35]. The tight relationship between climate and winemaking means that sector is one of the most susceptible to climate change [36], with air temperature and precipitation having a pivotal role in grapevine phenology [37,38,39], physiology [40,41], yield, and berry composition [27,42]. As the grapevine’s vegetative and reproductive cycles are tightly connected to the climatic conditions of each region, temperature and water availability play the biggest roles in this plant’s development and fruit quality [6,10].

## 3. Unveiling the Impact of Abiotic Stress on the Grapevine

The grapevine, like any other crop, relies heavily on temperature and water availability as crucial abiotic factors, with the first determining the duration of each phenological stage during the grapevine’s growth cycle [43,44]. It is also this relationship between temperature and phenology that leads to varieties being classified from early to late ripening, depending on their thermal requirement [45,46]. Precipitation is also constraining to grapevine growth, as the availability of water in the soil directly affects the plants’ water status [47]. However, as the grapevine is moderately tolerant to stress conditions, climate change effects need to be analysed as a whole, taking into account the conjunction of increased air temperature, lower water availability, and increased radiation and CO_2_ levels [48,49].

**Figure 1:**
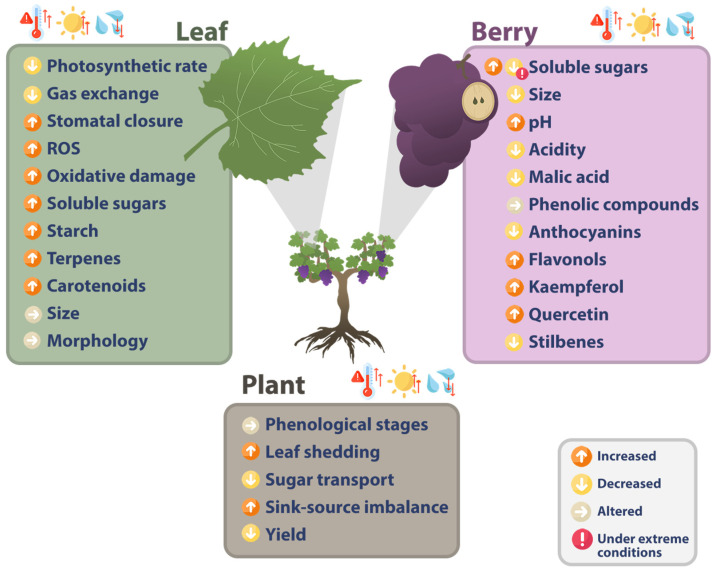
Summary of the effects of high temperature, radiation and drought on different parts of the grapevine.

Evidence heavily suggests that water stress, higher temperatures and increased radiation have different effects on the grapevine depending on the region, though the general agreement is that grapevine growth and development are impaired and that there is a general decrease in grape quality [30,49,50]. These abiotic factors trigger several biological responses from the plant, which can impact their survivability, quality and overall productivity (summarized in Figure 1) [13,36].

### 3.1. Summer Stress and the Major Constraints for Grapevine

As previously mentioned, temperatures are expected to rise in every predicted climate change scenario [32]. In recent years, advancements in the grapevine phenological stages have been extensively reported due to the increasing temperatures [29,31,36,51,52,53,54]. In fact, a number of studies have evaluated the impact of the century-long rise in temperature and predicted future trends [28,29,36,55,56], concluding that climate change could shorten the growing season by up to a month with the advancement of the bud break and flowering periods, causing the maturation period to occur during the hottest periods [36,53,56,57]. Additionally, higher temperatures during budburst could also lead to reduce flowering, while at the flowering stage they may cause flower abscission, leading to a decrease in plant yield [58,59]. These phenomena are expected to affect wine typicity due to the altered properties of the berries, resulting in wines with high alcoholic content and lower acidity, as well as atypical aroma and colour [44]. Earlier phenophases have been reported over the past years in several winegrowing regions, especially those of the Old World, such as France [52,60], Italy [61,62], Germany [63,64], and Portugal [40,65,66,67]. Higher temperatures, namely during the grapevine’s winter dormancy period, alter the timing of phenological stages. This is due to a lack of chilling accumulation, which negatively impacts bud break [61]. This increase in temperature will most likely defy the ability of the older regions to produce quality grapes and wines, especially in combination with lower water availability [12,68]. Furthermore, prolonged periods of summer stress during grape ripening are frequently reported as negatively influencing the quality and composition of the grapes, and thus of wine [69].

It is therefore important to understand how grapevine physiology is affected by high temperatures and drought periods, and how it affects the different organs of the plant.

### 3.2. The Impact of Water Stress and High Temperatures on Grapevine Leaves’ Physiological and Biochemical Stress Markers

While most of the research focuses on the effects of high temperatures and water stress on grape quality, it is crucial to delve into the leaf-level dynamics in order to gain fundamental insights for summer stress tolerance. The leaves serve as important interfaces between the plant and the environment, functioning as photosynthetic organs, promoting light interception, hydraulic constraints, gas exchange, and thermoregulation [70,71]. When these organs are subjected to high temperatures, photosynthesis is one of the first biological processes to undergo inhibition because of its extreme heat sensitivity [72,73], with this inhibition being one of the most evident effects observed in grapevines under summer stress [13]. In fact, as temperatures rise above 35 °C, the electron transport rate of photosystem II (PSII) is severely affected, leading to an increase in non-photochemical quenching (NPQ) as a defensive response and a safeguard measure of grapevine leaves against excessive radiation [74]. At higher temperatures, specifically above 40 °C, grapevine leaves face damage to their PSII, particularly if this stress is sustained for an extended period [75]. When temperatures exceed the 45 °C mark, the photosynthetic rate of the leaves diminishes considerably, something which is not only attributed to stomatal closure [76] but also to the inactivation of ribulose-1,5-biphosphate carboxylase oxygenase (RuBisCO) [77]. However, grapevines grown in Australia are constantly subjected to periods of temperatures above 40 °C during their growth and development [78] and, despite the aforementioned RuBisCO limitations, the effects of this exposure appear to be different between varieties [79]. In fact, this can also be attributed to differences in canopy temperature, which has been observed to vary between varieties grown under the same conditions [80].

Another limiting factor to photosynthesis in grapevines under abiotic stress is RuBisCO activity, which has been observed to be reduced due to heat stress, along with photosystem II (PSII) activity [81], while having reduced regeneration capacity under severe drought [77]. Though water stress is in part responsible for decreased photosynthetic activity in this species, this is mostly due to the plant preventing water loss through stomatal closure, leading to reductions in gas exchange rates [82,83,84]. Moreover, the combination of drought and heat stress is well known to lead to leaf wilting, impaired plant development, and, ultimately, reduced grape productivity and quality [85,86].

As the grapevine endures environmental stresses, other pathways become affected, while some are stimulated to help mitigate the negative effects. Changes in redox balance, such as increases in reactive oxygen species (ROS) concentration, are reported throughout the literature for summer stress, and are in part associated with the sensitivity of PSII to temperature [87]. Oxidative stress homeostasis in grapevine leaves is a complex system, involving ROS themselves, enzymes, hormones, and antioxidant compounds [88]. In fact, ROS are normally produced under various metabolic processes, but, as expected, increase drastically under stress conditions [88]. This leads to oxidative stress, which affects important leaf and berry metabolic processes, enzymatic activity, gene regulation, and even cause oxidative damage in cell membranes, leading to cell death on several [89]. An example is carbohydrates, which are found to increase in grapevine leaf when under heat stress [90]. Starch and soluble sugars are synthesized during photosynthesis, being used to store energy, produce organic compounds, and as the building blocks of cellulose and hemicellulose [91]. However, higher temperatures affect their partitioning, leading to accumulation in grapevine leaves [59,92], especially during night time, as warmer temperatures hinder their transport to the berries [92,93]. This increase is also theorized to be responsible for affecting the photosynthetic activity of grapevines during the day due to mechanisms of end product feedback downregulation [92]. Other metabolites, such as volatile compounds like terpenes and carotenoids, seemingly increase in concentration when these plants are under heat stress [94], having been attributed several protective properties, including the alleviation of the aforementioned oxidative stress [95,96]. In fact, previous studies have observed that grapevine plants capable of releasing monoterpenes are able to maintain higher photosynthetic rates and stomatal conductance [94], while carotenoids have been described as alleviators of the effects of heat stress, acting as quenchers of chlorophyll molecules and as direct scavengers of ROS, with their action being dependent on the grapevine variety [97,98,99].

Leaf anatomy has also been revealed to be modulated in the process of acclimatization to climatic conditions [100], with some studies hypothesizing that the number of leaves and the leaf size of a grapevine plant are not only influenced by the conditions of the growing season, but also by the climatic conditions endured on the previous year [101,102]. Several leaf parameters have been previously studied in the context of climatic influence; however, in-depth analysis of leaf parameters and intraspecific diversity in grapevine are still scarce. These studies could be important in terms of the understanding of varietal adaptability. For instance, stomata in grapevine leaves are known to be highly influenced by environmental factors, including radiation, air temperature, humidity, and the concentration of atmospheric CO_2_ [103]. Furthermore, stomatal density is usually influenced by the growing condition of the plant, with smaller stomata and/or higher stomatal density seemingly reducing transpiration, a possible adaptation to water stress [104,105]. Despite stomata density being previously associated with varietal differences, recent studies have shown this morphological parameter to present some plasticity, being mostly influenced by environmental conditions [84,104,106,107]. Understanding if grapevine varieties possess different plasticity regarding some of these characteristics can actually aid on improving the adaptability of the culture to climate change. This also includes other leaf cell parameters, such as cell thickness and the waxy cuticle layer of the leaf, which have been associated with protection against dehydration, UV radiation, and pathogen infection [108,109]. This is mainly attributed to the waxy cuticle, a thin layer of wax composed of alkanes, alcohols, and esters, which covers the surface of plant leaves as well as the outer surface of the grape. This cuticle is crucial in fruit protection, shielding the plant organs from the environmental conditions, with its thickness, structure, shape, and chemical composition influencing the impermeability of the leaf and the berry, and, thus, the ability of the plant to retain water and nutrients [109].

Lastly, and despite the effects of the aforementioned climatic impacts, the increase in CO_2_ concentration in the atmosphere would be expected to have a positive effect in grapevine growth and development, as it is the elementary molecule in the origin of plant biomass [110]. In fact, the high concentration of CO_2_ in the atmosphere might increase the photosynthetic rate, water use efficiency, and vegetative growth of these plants [76]. However, as both the changes in temperature and precipitation are expected to have negative effects, the interaction of all of these changes might not be beneficial to the plant. By understanding the effects of summer stress in the biological processes and anatomy of the leaf we can better comprehend the plant’s response to environmental challenges, as well as other phenomena such as leaf shedding [109]. These metabolic changes in the leaf are known to affect grape quality, as source–sink relationships are affected under stress conditions and can lead to imbalances and abnormal berry development. Thus, it is important to consider how fruit parameters and quality are influenced by the environment.

### 3.3. Climate Change Associated Effects on Grape Quality

Evidence heavily suggests that extreme temperatures and long periods of drought lead to a general decrease in grape quality, with smaller berry size and weight, and altered biochemical properties [30,49,50]. However, changes in grape berry composition due to abiotic stress are vast, and far from being fully understood [111]. Grape berries are composed of water, sugars, nitrogen compounds, organic acids, minerals, phenolics, and aromatic compounds [112]. It is the differences in concentration and quality of these compounds that partially dictate wine typicity, as they affect the flavour, aroma, colour, and overall character of each wine [26]. Thus, the ripening process and sugar accumulation are essential for grape berry quality, and both are accelerated by exposure to high temperatures [69,113], or even higher atmospheric CO_2_ levels [114]. Nonetheless, despite the grapevine’s capability of enduring stress, this is only true up to a certain threshold in temperature, with extreme and prolonged periods of heat harming both processes [111,115]. In fact, grapevines subjected to higher temperatures during the night usually have their sugar transportation mechanisms impaired [92], and as a sustained influx of sugar is extremely important for cell division, cell expansion and ripening, this reduced concentration severely affects the quality and the development of the berry [113]. This is one of the reasons why berry size usually reduces if prolonged and intense periods of summer stress occur during the veraison to maturity stages [114]. In the same manner, increased severity and prolonged periods of drought lead to lower yields and smaller, despite some level of water deficit being necessary to improve fruit quality [85]. Accompanying these changes in plant yield and berry size are alterations in biochemical composition of the fruit.

Of the compounds present in grape berry, organic acids, including tartaric acid and malic acid, are what characterize the fruit titratable acidity and the acidic harmony of the wines produced [116], and, similarly to sugar metabolism and transport, their metabolism is also affected by high temperatures [111]. Malic acid and tartaric acid are both synthetized in the early phases of berry development, and their concentration in grape berry is variable [117,118]. Malic acid is usually found in lower quantities, due to its degradation by the enzymatic action of malic enzymes, a process known as malic acid respiration [119], which is increased under heat stress [118]. Meanwhile, tartaric acid concentration seems to be extremely stable and genotype dependent [117,120], with few studies mentioning environmental influence [121,122]. Besides organic acids, potassium also plays a pivotal role in grape acidity and pH, increasing in concentration under higher temperature and water stress, which leads to higher pH levels [123]. As pointed out by Duchêne et al. [117], and given the importance of grape acidity in the wine production, understanding the behaviour of grapevine varieties regarding these organic acids, could be a key component in the adaptation to climate change.

Phenolics, such as flavonols, stilbenes, phenolic acids, and anthocyanins, are some of the most important secondary compounds in grape berry, with their synthesis being mostly affected by higher temperatures and radiation [111,124]. These compounds are responsible for the colour, aroma and flavour of grapes and wines, while also being attributed several health benefits [125]. For instance, flavonols, including catechins, proanthocyanidins, quercetin and kaempferol, are partly responsible for the antioxidant properties observed in grape products, as well as being associated with the bitterness and astringency of wines [126]. In terms of grapevine tolerance to stress, at the berry level this class of flavonoids is associated with protection against UV radiation [127], with their synthesis being stimulated under higher temperatures. Nonetheless, long-term exposure leads to their breakdown and reduced concentration in the grape berry [128,129]. Other phenolic compounds, namely quercetin and kaempferol, are important in the yellow coloration of white grape varieties, similar to how anthocyanins are responsible for the typical shades of red, purple, and blue in red grape varieties [130,131]. As colour is actually one of the most important sensory properties in wines, especially to consumers, their concentration an extractability dictates the hue of wines [132,133,134]. Anthocyanins are actually sensitive to high temperature, which accelerates their degradation leading to lower concentrations, and in turn affecting wine colour intensity and stability [114,135,136,137,138], despite some studies pointing towards increased anthocyanin biosynthesis under stress conditions [127,139].

Lastly, of stilbenes compounds in grape berry, resveratrol is usually highly mentioned due to its potential health benefits [140,141]. Similarly to the aforementioned phenolic compounds, high temperatures have been shown to negatively influence their concentration, decreasing progressively under high temperatures [142,143], and being inversely proportional to anthocyanin concentration [136]. However, these might play some kind of role under drought, as studies of the influence of water deficit in the stilbene biosynthesis have shown these compounds to accumulate under these stress conditions [144,145].

In order to mitigate the aforementioned effects in the grapevine, and given the importance of vitiviniculture in the economy of several countries, it is essential to develop new and improved adaptation strategies. Over the past years, research has focused on different adaptation strategies by which to provide viticulturists and winemakers with accessible tools for mitigating the negative effects of climate change [2,12,13,14,31,146,147,148,149], while also taking into consideration the sustainability of its processes and accounting for the producers’ and the consumers’ points of view [133,150,151].

## 4. Adaptation Strategies Amidst Climate Change

**Figure 2:**
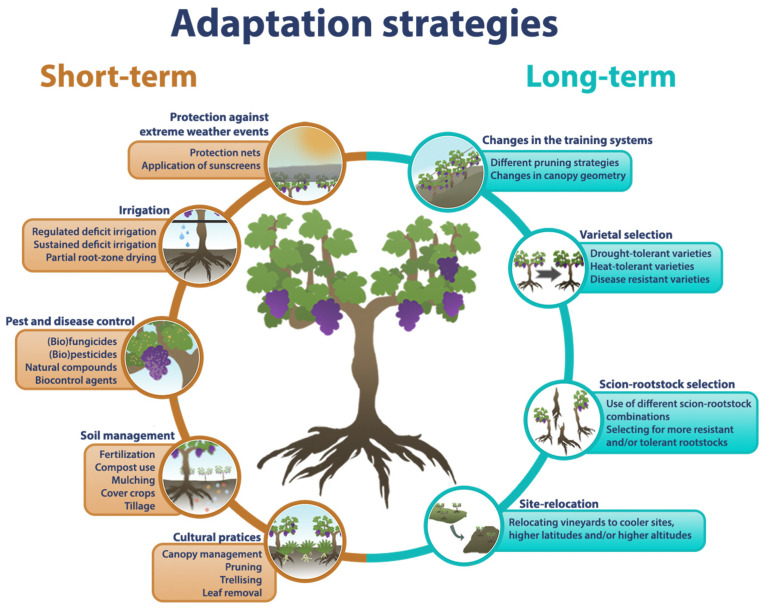
Infographic summarizing the different climate change adaptation strategies available for viticulture, classified as short term (orange) or long term (blue).

With regards to viticulture and climate change, adaptation strategies are defined as sets of actions, processes and approaches that aim at reducing the negative effects of climate change [152]. These strategies have been the focus of intensive research over the past years [13,45,110,147,148,149,152,153], with most authors dividing them into two categories, short-term or long-term (summarized in Figure 2), solely based on when the change can be implemented, with short-term strategies being applicable during the growing season and long-term strategies requiring more invasive procedures in the vineyard [14,147].

Short-term strategies are the most common, as they do not require substantial interventions in the vineyard, while also being flexible, allowing for adaptation to yearly conditions [147]. These include cultural practices, protection against extreme weather events, irrigation, pest and disease control, and soil management [147]. Cultural practices consist mostly of canopy management techniques, such as pruning, trellising, removing leaves, or altering shoots, and have been used for centuries in viticulture, aiming at improving grapevine productivity and berry quality [154,155,156]. In a similar way, irrigation, pest and disease control, and soil management are strategies that are becoming increasingly needed, and have even been established as mandatory for a good production [157,158,159,160,161,162]. Moreover, new products and techniques have been developed over the past years to aid with protection/adaptation to extreme weather events and abiotic stresses, such as protective covers [163] and protective films [164,165].

Unlike short-term adaptation strategies, long-term ones require more invasive procedures, encompassing changes in the training system, varieties used, scion–rootstock combinations, and even whole vineyard relocations. These strategies, despite being considered more sustainable in the long run, call for greater initial investment, major changes in agricultural practices, and time, making viticulturists more hesitant on their implementation [14]. This group of strategies is often deemed more effective in the adaptation to climate change, but the higher demand in time and financing make their study mor challenging. Nonetheless, recent literature regarding this topic has emerged. A more intensive strategy is vineyard site relocation, which is frequently mentioned in review articles [12,14,47], but is mostly a last resort strategy for regions where viticulture is truly threatened, with its viability and success depending on several factors. In contrast, the training system, which dictates grape production and quality [166], could be adapted to increase drought resistance, delay phenology, optimise canopy geometry, and even alter leaf and bunch microclimates [12,14,47]. In the same manner, scion–rootstock selection is also promising, as changing the rootstock used can lead to increased grapevine tolerance to abiotic stress, while maintaining local wine typicity [147,167,168]. In fact, this approach has been practiced throughout the history of viticulture with the selection of more drought-tolerant rootstocks [168], while scion selection has been typically focused on disease resistance, yield, and quality [169]. Nonetheless, varietal selection aims to go a little further, basing itself on the adaptability differences of grapevine varieties [18,170]. These characteristics can range from phenological timing to abiotic stress tolerance, while also encompassing the development of new genotypes [170].

Selecting and implementing an adaptation measure in a given vineyard is an elaborate process which requires determining costs and benefits. Furthermore, and despite the capability of these strategies, it is still predicted that some of the negative impacts of climate change will remain noticeable in viticulture [146]. Moreover, as the effectiveness of each strategy is difficult to assess over different regions and growing conditions, there is an increasing need in the variability of studies for each one.

Increasing viticulture resilience by using the already available intraspecific crop diversity can be a promising adaptation strategy. Given the traditional practices and local regulations, along with the time taken and financial costs associated with the replantation of vineyards, changing cultivated grapevine varieties might not be the first choice of producers, who prefer faster results [17]. However, and given the previous work done over decades, varietal selection is seemingly a robust long-term adaptation strategy.

## 5. Varietal Selection in Viticulture—Steps Towards the Understanding of Adaptation

The urgency for climate change adaptation has led to increased research surrounding adaptation strategies for viticulture, including studies on the intraspecific diversity of grapevine [17,171,172,173]. Studying the genetic diversity of grapevine varieties and their overall behaviour to environmental conditions can in fact lead us to either finding varieties better prepared for the predicted climate change scenarios or varieties that possess the desired traits for new crossing projects [17].

It is well established that the phenology of grapevine is highly affected by the climate, and tied to the genome, which affects sugar and organic acids metabolism [174]. Consequently, one of the most common suggestions in varietal selection involves the use of late ripening varieties [13,16], as the advancement of the phenological stages will lead to the maturation occurring during warmer conditions [29,52,66]. However, these might become unreliable in the future, as plant phenology is guided by various polygenetic traits [175,176] and yearly environmental conditions [11,67,177]. In fact, fluctuations in the amount and timing of rainfall, as well as the frequency and intensity of extreme temperature events, from one year to another and from one location to another is substantial in several agricultural regions and may be intensifying due to climate change [67,178]. However, cultivar turnover is projected to be able to decrease the loss of agricultural areas by over 50% in a 2 °C warming scenario, reducing the negative impacts of climate change in viticulture, especially in warmer countries which might need to implement more than one mitigation measure in order to prevent losses [17]. This turnover is based on the premise of varietal selection, where grapevine varieties could be selected according to their adaptation capabilities and used in different combinations of scion × rootstock × training system.

### 5.1. Grapevine Varieties and Their Distinctive Adaptation Strategies

As mentioned earlier, long-term adaptation strategies, such as using optimally adapted grapevine varieties to a given region, might be one of the best measures to implement and to increase the sustainability of this agricultural sector [179]. Despite being the same species, grapevine varieties still present a lot of genomic and phenotypic plasticity [180,181]. This high diversity is especially observed in *Vitis vinifera*, as years of artificial selection among viticulturists has led to plants with specific traits and adaptative mechanisms to assist with growing in unfavourable conditions [182,183].

The leaves are the interface between the plant and the environment, acting as photosynthetic organs, and promoting light interception, hydraulic constraints, gas exchange and thermoregulation [70,71]. Morphoanatomical differences can be observed in the leaves of different grapevine varieties, not only in leaf size and shape but also in epidermal cells, stomata number and morphology and cuticle thickness [100,183]. Environmental conditions have actually been shown to modulate the development of the leaf in several *Vitis* species [102], despite the complex genetic architecture associated with it [184]. In fact, the morphoanatomy of the leaf is modulated by the environmental conditions, being especially evident if the plant is subjected to drought [185,186,187], and increased temperature and radiation [70,72,188,189,190]. However, the response and even the adaptability might differ between varieties. For instance, Teixeira et al. [105] analysed six Portuguese white varieties grown under the same conditions, concluding that genotypes with smaller leaves, higher leaf density and higher stomata density, such as those of cv. Viosinho, were possibly better adapted to drier and warmer climates. Another work with red varieties also led to similar conclusions, proposing that cvs. Trincadeira, Cabernet Sauvignon, and Syrah could have a comparative advantage to sustaining abiotic stresses [191]. Beyond this, leaf stomata has also been observed to vary in architecture and density between varieties of *V. vinifera* [192,193]. Despite there being a lack of studies surrounding this topic in grapevines, stomata play a crucial role in water loss, while also being highly regulated by environmental conditions [194]. Grapevine leaves only present these structures in the lower epidermis, which aids in decreasing water loss by transpiration. However, as the size, width, and length of the stomata differ between varieties [192], so does their capability of withstanding longer periods of abiotic stress. In fact, smaller and more dispersed stomata have been correlated to lower transpirations rates, while longer and wider stomata increase response plasticity under summer stress [105,195]. Moreover, though these structures have barely been previously studied in grapevine, stomata were previously observed to be rearranged to optimize stomatal conductance, and even decrease in size in order to improve WUE in other species [194]. Along with these differences in morphoanatomy, the regulation of leaves also varies among grapevine varieties, and physiological parameters, such as stomatal conductance, photosynthetic rate and transpiration rates differ between varieties under the same conditions [103,196,197,198,199,200]. For instance, Vaz et al. [201] observed that cv. Tempranillo and cv. Trincadeira, despite having similar leaf area, behaved differently under drought, contrasting in leaf water potential, stomatal conductance and reflectance. The same has been observed in other red grapevine varieties, where cv. Touriga Franca and cv. Syrah, despite being well adapted to warmer conditions, behaved differently depending on the soil water availability [107]. Another study with cv. Semillon and cv. Muscat Blanc à Petits Grain grown under the same pedo-climatic conditions also hypothesized cv. Muscat Blanc à Petits Grain to be better adapted to abiotic stress due to higher CO_2_ assimilation rate, photosynthetic pigment concentration and midday leaf water potential [202]. These differences in response to water availability, specially under drought conditions, have led authors to classify each variety as isohydric, plants who close their stomata when soil water potential drops, or anisohydric, those who continue to transpire despite the decrease in soil water potential [203,204]. Nonetheless, this classification is plant-environment dependant in the case of grapevine, as, despite having a tight stomatal control, the same variety can present different hydraulic strategies, with the environmental conditions of its development dictating this behaviour [193,203,205,206,207].

Grapevine varieties differ in mesophyll thickness, trichome density, leaf area, and canopy architecture, despite the lack of scientific literature on this matter [179], as well as in the concentration of photosynthetic pigments, chlorophyll a, chlorophyll b, and carotenoids. This variability in photosynthetic pigments might be associated with tolerance to abiotic stress, especially as certain varieties rely on carotenoids to scavenge ROS [98]. Moutinho-Pereira et al. [208] observed that Portuguese variety cv. Tinto Cão presented a different ratio of chlorophyll pigments and concluded that it could be an adaptation strategy to both higher radiation levels and the combination of increased air temperature with drought. In fact, other studies with heat, water, and light stresses, revealed that cv. Touriga Nacional and cv. Trincadeira present different contents of photosynthetic pigments, highlighting the contrasting behaviour of different varieties [98]. As varieties differ in what is arguably the most important component of leaves, this can indicate intrinsic varietal behaviour that can lead to different adaptability.

With regard to tolerance traits in the grape berry, most of the studies focus on the morphoanatomical traits and resistance to pathogens, with very little attention being given to abiotic stress. In fact, the abiotic stress tolerance mechanisms of berries is still quite understudied [209]. Nevertheless, recent research has focused on the influence of water deficit in the cuticular waxes of the grape berry, and has observed a correlation between the increase in stress with increasing wax content and the upregulation of several candidate genes of the wax biosynthetic pathway [210]. In fact, Hewitt et al. [211] have shown that berries of different varieties do in fact respond differently to the same stress. In their study with cv. Cabernet Sauvignon and cv. Riesling after exposure to heat and water stress, these authors observed that both varieties activated different genetic mechanisms, despite resulting in a similar physiological outcome [211].

Although promising, these studies (summarized in Table 1) mostly reflect the behaviour of these varieties to a certain stress and could very well be completely different under other environmental conditions. Therefore, it is important to infer what molecular mechanisms are underlying these responses.

### 5.2. Exploring the Molecular Basis of Stress Resilience in Grapevine

Genomics, transcriptomics, and proteomics are powerful tools for assessing varietal differences. Although genome regions associated with certain climate- and environment-smart traits have been identified, polymorphisms from years of varietal crossing remain largely unknown. With the advancements in genomic tools over the past years, research of the molecular mechanisms that underline acclimation and adaptation processes has been progressing steadily, despite phenotype and adaptation being highly polygenic. The access to new technology has allowed research to demonstrate that it is possible to distinguish the transcriptome of different grapevine varieties [98,173,179,214]. Recent research with native Portuguese varieties has unveiled how using molecular techniques can be extremely important in the near future: a gene array has been developed by comparing leaves of cv. Touriga Nacional, which was considered better adapted to extreme conditions, and cv. Trincadeira [214]. Both varieties were subjected to individual and different combinations of stresses, such as lack of irrigation, high radiation, and heat; with each transcriptomic response being analysed. Following this study, Carvalho et al. [173] designed a custom quantitative reverse transcription polymerase chain reaction (RT-qPCR) array with 65 differentially expressed genes in cv. Antão Vaz, cv. Bastardo, cv. Castelão, cv. Cerceal Branco, cv. Encruzado, cv. Fernão Pires cv. Moscatel Graúdo, cv. Tinta Barroca, cv. Touriga Franca and cv. Viosinho. Both of these studies led to an assay on the tolerance of several red and white Portuguese varieties to abiotic stress, with the outcome being the categorization of nine varieties into tolerant or sensitive [98,173,214]. However, similar studies regarding gene expression and abiotic stress response in grapevine are still somewhat scarce, despite gene technology becoming increasingly less expensive and more accessible. This can partially be attributed to some traits being highly polygenic, making it hard to pinpoint exact pathways or genes. Nonetheless, over the past years, significant progress has been made in understanding the molecular basis behind abiotic stress response and adaptation.

As previously mentioned, grapevines under abiotic stress usually produce berries with a higher content of anthocyanins and other phenolics. In fact, berries of grapevines under water deficit reveal a higher content of phenolics, which has been associated with increased activity of the anthocyanin biosynthesis gene *VvUFGT*, as well as genes related to the flavonoid pathways *VvCHS2*, *VvCHS3,* and *VvF3H* [139]. Matus et al. [215] also observed that several *VvMYBA* genes, transcription factors of the *UFGT* gene, were UV sensitive, leading to the accumulation of anthocyanins in plant organs. An increase in anthocyanins under stress conditions can be attributed to several protective properties, especially against abiotic stresses such as excessive solar and UV radiation, ROS scavenging, or even in signalling cascades [216,217]. Despite that, this behaviour might not be as linear as previously thought, as it can differ between varieties. For instance, under high temperatures cv. Sangiovese was shown to have reduced anthocyanin content, which was associated with the overexpression of the peroxidase gene *VviPrx31* and the downregulation of *VvUFGT* and its precursor *VvMYBA*, as well as flavonoid biosynthesis genes *VvF3′5′Hi* and *VvDFR* [218]. Increased temperatures have also been observed to disrupt the anthocyanin/sugar ratio, leading to lower anthocyanin content and higher amounts of soluble sugars; however this seems to be highly varietal-, and even clonal-, dependant [219].

Soluble sugars are known to accumulate in grapevine organs, with high temperature possibly playing a pivotal role in the associated genes. For example, galactinol has been shown to accumulate in grape berries of cv. Cabernet Sauvignon under heat stress, which has been attributed to the overexpression of the genes *VvGOLS1* and *VvHsfA2* [220]. Similarly, under water deficit conditions, sugar transporter genes *VvHT1*, *VvHT5* and *VvSUC11*, as well as *VvMSA*, the grapevine ABA stress and ripening-induced (ASR) protein, were observed as being differently expressed, indicating a pivotal role in stress tolerance response [221]. Analogously, *VvSUC11*, *VvSUC12* and *VvSUC27* were recently observed to play a regulatory role in several types of abiotic stress, being upregulated under different scenarios [222].

Other genes, such as those related to heat-shock proteins (HSPs), aquaporins, ROS metabolism, chlorophyll synthesis and berry ripening are also key factors in the defence against abiotic stress [223,224]. For instance, the synthesis of HSPs and heat-shock transcription factors (HSFs) is typically increased under heat stress, as these are involved in the protection of the photochemical reactions in PSII and protein folding and denaturation, as well as aiding in maintaining cellular homeostasis and in the response to oxidative stress [225,226]. Zha et al. [87] have observed that some of these genes, including *VvHSFA2*, *VvHSFA7* and *VvsHSP*, are differently expressed between heat-tolerant and sensitive varieties, indicating differences in stress response. On another note, and besides the aforementioned functions, *VvHSP20* genes have also been correlated with a putative protective function during berry ripening, indicating an important role that these proteins may have during berry development under stress conditions [227].

Aquaporins, proteins responsible for facilitating the transfer of water and small solutes across membranes, can also be linked to a better drought-stress response [228]. For example, Shelden et al. [229] have observed that cv. Chardonnay and cv. Grenache presented different water management strategies, with the latter being considered near-isohydric and presenting a tighter regulation of the aquaporin genes *VvPIP1;1*, *VvPIP2;1*, *VvPIP2;2*, *VvPIP2;3*, *VvTIP1;1*, and *VvTIP2;1* under stress. In fact, *VvPIP2;1* and *VvTIP2;1* gene regulation has been recently observed to differ slightly between different varieties under drought-stress conditions [230].

The waxy cuticles of both the leaves and the berries are also stress tolerance associated, with their associated genes being differently expressed under abiotic stress conditions. For instance, the *β-ketoacyl-CoA synthase* (*KCS*) genes of cv. Muscat Hamburg have shown high transcription levels in the leaves of water stressed plants, enhancing cuticular wax accumulation and reducing water loss [231]. The same has been observed for the berries of cv. Merlot, where, under water stress, cuticular wax content also increased, while genes of the aliphatic wax biosynthetic pathway *VvCER10*, *VvCER2*, *VvCER3*, *VvCER1*, *VvCER4*, and *VvWSD1* were upregulated [210].

A summary of the studied genes mentioned in this section is presented in Table 2. Despite all these studies, there are hundreds of different varieties, each with its own genotype and phenotype, with each interacting differently with its environment. Thus, it is necessary to couple several study areas such as plant physiology, biochemistry, histology, and genetics, in order to better understand each variety’s capability of adaptation.

## 6. Conclusions and Future Prospects

Climate change poses significant challenges to viticulture worldwide, especially in the Mediterranean Basin, impacting both grape production and wine quality. Addressing these challenges often relies on short-term strategies, such as irrigation, which, despite their increasing use, raise concerns regarding their long-term viability. Given the remarkable intervarietal diversity of grapevines, varietal selection emerges as one of the most promising long-term adaptation strategies. In fact, and as highlighted in this literature review, grapevine varieties can differ in stress response, indicating distinct tolerance and adaptability, which could be leveraged to improve the sustainability of viticulture. Nonetheless, research on this topic is still limited, with many varieties remaining understudied compared to the more widely recognized ones. This overlooks the potential of autochthonous and underutilized varieties, which may possess unique traits better suited to predicted climate change scenarios. Therefore, future research should prioritize identifying and characterizing grapevine varieties with high adaptability to abiotic stresses, particularly heat and drought tolerance. Moreover, field trials under natural terroir conditions, exposing grapevines to temperature, precipitation, sun exposure, and cultural practices, could provide more accurate insights into varietal adaptability and suitability to specific regions, as controlled experimental set-ups are often not enough to mimic the complexity of environmental variables and their added effects. Collaborative efforts pooling data from these field trials could enhance varietal classification and uncover additional molecular responses critical for adaptation, enhancing the applicability of the varietal selection.

Varietal selection could be especially important in regions with rich inter- and intravarietal diversity, such as Portugal. Despite its extensive diversity, few Portuguese varieties have been studied thoroughly. By focusing research on less prominent varieties, this could serve as a case study for implementing varietal selection strategies, offering replicable models for other countries, especially those in the Mediterranean Basin.

Expanding research on varietal selection is a promising pathway for future climate change adaptation. Highlighting the existence of varieties that seem better adapted to specific conditions reinforces the potential benefits of this approach, even as significant knowledge gaps remain. Despite the progress made, hundreds of grapevine varieties, each with unique genotypes and phenotypes, interact differently with their environments. Thus, coupling several areas of study, such as plant physiology, biochemistry, histology, and genetics, is crucial to better understand each variety’s capacity for adaptation. The focus should not only be on the most cultivated varieties but also on the understudied ones. Moreover, understanding the processes behind varietal adaptability will not only improve varietal selection, but also breeding programs.

Finally, by combining efforts and enhancing comparative varietal studies, the viticulture sector can truly benefit from varietal selection, ensuring the resilience and sustainability of global viticulture in the face of an increasingly challenging climate.

## Figures and Tables

**Table 1:** Studies assessing differences in the response/tolerance of different grapevine varieties to abiotic stress.

Variety	Berry Colour	Country	Growth Conditions	Type of Stress	Observations	Tolerance to Stress	Reference
Albarin Blanco	White	Spain	Field trial	Summer stress	Medium stomatal length, width, and density.	Unconclusive	[192]
Alvarinho	White	Spain	Field trial	Summer stress	Medium stomatal length, width, and density.	Unconclusive	[192]
Alvarinho	White	Portugal	Field trial	Summer stress	Smaller leaves, epidermal cells with intermediate thickness, thinner waxy cuticle. Lower stomatal density.	Sensitive	[105]
Alicante Bouschet	Red	Spain	Field trial	Summer stress	Medium stomatal length, width, and density.	Unconclusive	[192]
Antão Vaz	Red	Portugal	Field trial	Summer stress	Response varied between different field conditions, being sensitive to light stress, and moderately sensitive to drought and heat stresses in one, while being sensitive to light, drought, and heat under harsher conditions.	Sensitive	[173]
Aragonez	Red	Portugal	Field trial	Drought	Higher leaf temperature, lower stomatal conductance, gradual decrease of water potential, lower water use efficiency, lower net photosynthetic values.	Unconclusive	[107]
Aragonez	Red	Portugal	Field trial	Drought	Drought regime led to decreased stomatal conductance. Higher values of total leaf chlorophyll maintained during drought. Reduced maximum assimilation rate, maximum and apparent quantum yield. Increased reflectance under drought. Decreased brix.	Less tolerant	[201]
Aragonez	Red	Portugal	Field trial	Summer stress	Highest chlorophyll and carotenoid content, lowest net photosynthetic rate, lowest soluble sugar and starch content, lower net photosynthetic rate, lowest stomatal conductance.	Less tolerant	[76]
Aragonez	Red	Portugal	Field trial	Summer stress	Higher concentration of chlorophylls and photochemical response, low stem water potential, low values of stomatal conductance and net CO_2_ assimilation rate.	Tolerant	[212]
Aragonez	Red	Spain	Field trial	Drought	High concentration of photosynthetic pigments and high values of photosynthetic parameters, low intrinsic water use efficiency, higher stomatal conductance, and net CO_2_ assimilation.	Sensitive	[213]
Arcos	Red	Spain	Field trial	Summer stress	Lower stomatal conductance values and is considered one of the most stressed varieties. High stomatal density coupled with lower stomatal dimensions.	Tolerant	[200]
Argamussa	White	Spain	Field trial	Drought	Under progressive water depletion presented the maximum intrinsic water use efficiency.	Tolerant	[213]
Arinto	White	Portugal	Field trial	Summer stress	Larger leaves, epidermal cells with intermediate thickness, thinner waxy cuticle. Lower stomatal density.	Sensitive	[105]
Bastardo	Red	Portugal	Field trial	Summer stress	Overall tolerant to light, drought, and heat stresses. Response varied between different field conditions.	Unconclusive	[173]
Bobal	Red	Spain	Field trial	Summer stress	Higher stomatal conductance values, high intrinsic water use efficiency.	Tolerant	[200]
Cabernet Sauvignon	Red	Chile	Pots, open-air	Drought	High stomatal sensitivity to water deficit, lower reductions in photorespiration.	Sensitive	[197]
Cabernet Sauvignon	Red	Portugal	Field trial	Summer stress	Smaller leaves, high leaf density, and small and/or sunken stomata.	Tolerant	[191]
Cabernet Sauvignon	Red	Portugal	Field trial	Drought	Medium leaf temperature, lowest water use efficiency, highest number of stomata, highest specific leaf area.	Unconclusive	[107]
Cabernet Sauvignon	Red	Spain	Field trial	Drought	Low minimum stem water potential values, low intrinsic water use efficiency, tight control of stomatal aperture.	Sensitive	[213]
Cabernet Sauvignon	Red	Spain	Field trial	Summer stress	Medium stomatal length, width, and density.	Unconclusive	[192]
Cabernet Sauvignon	Red	USA	Greenhouse/laboratory	Drought/leaf dehydration	Intermediate leaf water loss, medium stomatal density, low stomatal sensitivity to water loss.	Tolerant	[193]
Caiño Blanco	White	Spain	Field trial	Summer stress	Medium stomatal length, width, and density.	Unconclusive	[192]
Caiño Tinto	Red	Spain	Field trial	Summer stress	Medium stomatal length, width, and density.	Unconclusive	[192]
Callet	Red	Spain	Field trial	Drought	Low stem water potential values.	Sensitive	[213]
Callet Blanc	White	Spain	Field trial	Drought	Low stem water potential values.	Sensitive	[213]
Carménère	Red	Chile	Pots, open-air	Drought	Lower stomatal sensitivity to water deficit, reduced in light.	Tolerant	[197]
Castañal	Red	Spain	Field trial	Summer stress	Low stomatal length, width, and density.	Unconclusive	[192]
Castelão	Red	Portugal	Field trial	Summer stress	Response varied between different field conditions. Sensitive to light, heat, and drought in one, and tolerant to heat and drought in another.	Unconclusive	[173]
Cerceal Branco	White	Portugal	Field trial	Summer stress	Tolerant to light and drought stresses and sensitive to heat stress under two different field conditions.	Tolerant	[173]
Chardonnay	White	Chile	Pots, open-air	Drought	Lower stomatal sensitivity to water deficit.	Tolerant	[197]
Chardonnay	White	Spain	Field trial	Drought	High minimum stem water potential values, low intrinsic water use efficiency.	Sensitive	[213]
Chasselas Dorée	White	Spain	Field trial	Summer stress	Medium stomatal length and width and with high stomatal density.	Unconclusive	[192]
Ekigaïna	Red	France	Field trial/greenhouse	Summer stress/drought	Isohydric behaviour, strongest stomatal response to changes in leaf water potential, reduction in fertility.	Sensitive	[196]
Encruzado	White	Portugal	Field trial	Summer stress	Medium-sized leaves, thicker upper epidermal cells and intermediate thickness of lower epidermal cells, intermediate waxy cuticle. High stomatal density.	Unconclusive	[105]
Encruzado	White	Portugal	Field trial	Summer stress	Tolerant to light and drought stresses and sensitive to heat stress in two different field conditions.	Tolerant	[173]
Escursac	Red	Spain	Field trial	Drought	Highest intrinsic water use efficiency, tight control of stomatal aperture.	Less tolerant	[213]
Esperó de Gall	Red	Spain	Field trial	Drought	Lowest leaf photosynthesis value.	Sensitive	[213]
Fernão Pires	White	Portugal	Field trial	Summer stress	Sensitive to heat, light, and drought stresses in both field conditions.	Sensitive	[173]
Forcallat	Red	Spain	Field trial	Summer stress	Lower stomatal conductance values and is considered one of the most stressed varieties. Highest intrinsic water use efficiency, high stomatal density coupled with lower stomatal dimensions. Berries with moderate total acid concentration and anthocyanin content.	Tolerant	[200]
Galmeter	Red	Spain	Field trial	Drought	High intrinsic water use efficiency, lowest stomatal conductance.	Sensitive	[213]
Garnacha	Red	Spain	Field trial	Summer stress	Higher stomatal conductance values.	Tolerant	[200]
Giró Ros	White	Spain	Field trial	Drought	Low stem water potential values, tight control of stomatal aperture. Under progressive water depletion presented the maximum intrinsic water use efficiency.	Tolerant	[213]
Godello	White	Spain	Field trial	Summer stress	Medium stomatal length, width and density.	Unconclusive	[192]
Gorgollasa	Red	Spain	Field trial	Drought	High intrinsic water use efficiency, low stem water potential values.	Sensitive	[213]
Grenache	Red	France	Field trial/greenhouse	Summer stress/drought	Isohydric behaviour; incomplete maturation.	Sensitive	[196]
Grenache	Red	Spain	Field trial	Drought	Moderate intrinsic water use efficiency and stomatal behaviour.	Sensitive	[213]
Grenache	Red	USA	Greenhouse/laboratory	Drought/leaf dehydration	Intermediate leaf water loss, high stomatal density, intermediate stomatal sensitivity to water loss.	Tolerant	[193]
Jacquez	Red	Spain	Field trial	Summer stress	Low stomatal length, width, and density.	Unconclusive	[192]
Macabeo	White	Portugal	Field trial	Summer stress	Medium-sized leaves with thicker upper and lower epidermal cells, thicker upper cuticle. High stomatal density.	Tolerant	Teixeira et al. (2018)
Macabeo	White	Spain	Field trial	Drought	Highest minimum stem water potential values, highest stomatal conductance, lowest intrinsic water use efficiency.	Sensitive	[213]
Malvasia de Banyalbufar	White	Spain	Field trial	Drought	Low stem water potential values.	Sensitive	[213]
Manto Negro	Red	Spain	Field trial	Drought	Tight control of stomatal aperture, low intrinsic water use efficiency.	Tolerant	[213]
Marselan	Red	France	Field trial/greenhouse	Summer stress/drought	Anisohydric behaviour. Maintained gas exchange under drought stress, complete maturation under severe water restriction.	Less tolerant	[196]
Mavrodafni	Red	Greece	Pots, sheltered	Drought	Steep decline in predawn water potential and lower values of stomatal conductance and photosynthetic rate. Highest leaf ABA concentration along with high pH values, promoting stomatal closure.	Less tolerant	[199]
Jaen	Red	Spain	Field trial	Summer stress	Medium stomatal length, width, and density.	Unconclusive	[192]
Merlot	Red	Spain	Field trial	Drought	Low stem water potential values.	Sensitive	[213]
Moll	White	Spain	Field trial	Drought	Tight control of stomatal aperture.	Sensitive	[213]
Monastrell	Red	Spain	Field trial	Summer stress	Higher stomatal conductance values, high intrinsic water use efficiency.	Tolerant	[200]
Moscatel Graúdo	White	Portugal	Field trial	Summer stress	Medium-sized leaves with high specific leaf area. Thinner upper epidermal cells and intermediate lower epidermal cells, thinner upper cuticle. High stomatal density.	Unconclusive	[105]
Moscatel Graúdo	White	Portugal	Field trial	Summer stress	Different response under different field conditions. Sensitive to heat, light and drought stresses in one, and only sensitive to drought in another.	Tolerant	[173]
Mourvèdre	Red	France	Field trial/greenhouse	Summer stress/drought	Isohydric behaviour, reduction in fertility.	Sensitive	[196]
Muscat Italia	White	Tunisia	Greenhouse	Heat	Increased leaf blade thickness, decreased palisade parenchyma thickness, folds in the adaxial surface. Elongated convex epidermal cells with less sinuous shape. Irregular giant pores on the adaxial surface. Chloroplasts suffered alterations in shape, thylakoid membrane orientation, grana stacking, starch granules and plastoglobuli.	Unconclusive	[189]
Muscat-à-Petits-Grains	White	Portugal	Field trial	Summer stress	Medium-sized leaves with thinner upper and lower epidermal cells, thinner upper cuticle. High stomatal density.	Unconclusive	[105]
Muscat-à-Petits-Grains	White	Portugal	Field trial	Summer stress	Higher midday leaf water potential, higher soluble sugars and lower total phenol concentration, higher efficiency of PSII, higher reflectance indexes, higher concentration of Ca^2+^ and Mg^2+^, higher stomatal density.	Tolerant	[202]
Razegui	Red	Tunisia	Greenhouse	Heat	Increased leaf blade thickness, decreased palisade parenchyma thickness, folds in the adaxial surface involving both cuticle and epidermal cells. Elongated convex epidermal cells with less sinuous shape. Irregular giant pores on the adaxial surface. Chloroplasts suffered alterations in shape, thylakoid membrane orientation, grana stacking, starch granules and plastoglobuli.	Unconclusive	[189]
Sabater	Red	Spain	Field trial	Drought	Tight control of stomatal aperture.	Sensitive	[213]
Savatiano	White	Greece	Pots, sheltered	Drought	Lower values of predawn water potential, stomatal conductance, and photosynthetic rate. Higher leaf ABA concentrations promoting stomatal closure at early stress stages.	Tolerant	[199]
Sauvignon Blanc	White	Chile	Pots, open-air	Drought	High stomatal sensitivity to water deficit, lower reductions in photorespiration.	Sensitive	[197]
Semillon	White	Portugal	Field trial	Summer stress	Lower midday water potential, lower stomatal conductance in the afternoon, higher non-photochemical quenching, higher concentration of K^+^, higher soluble sugar and lower photosynthetic pigments, higher total phenols concentration, higher thiobarbituric acid-reactive substance.	Sensitive	[202]
Shiraz	Red	USA	Greenhouse/pots/laboratory	Drought/leaf dehydration	Leaves lost the most water, highest rate of dehydration, lowest stomatal density, slow response to water loss via stomatal closure, stomata more sensitive to ABA application.	Sensitive	[193]
Syrah	Red	France	Field trial/greenhouse	Summer stress/drought	Anisohydric behaviour, maintained gas exchange under drought stress, complete maturation under severe water restriction.	Tolerant	[196]
Syrah	Red	Portugal	Field trial	Summer stress	High leaf density, and small and/or sunken stomata.	Tolerant	[191]
Syrah	Red	Portugal	Field trial	Drought (regulated deficit irrigation)	Highest leaf temperature, lowest stomatal conductance, highest water use efficiency, lowest number of stomata.	Unconclusive	[107]
Syrah	Red	Spain	Field trial	Drought	Low stem water potential values, low intrinsic water use efficiency.	Sensitive	[213]
Tinta Barroca	Red	Portugal	Field trial	Summer stress	Sensitive to light stress, being consistent in two different locations.	Sensitive	[173]
Tinto Cão	Red	Portugal	Field trial	Summer stress	Lower chlorophyll and carotenoid content but higher Chl a/b ratio, highest starch content, higher R:FR transmittance and reflectance, lowest leaf water potential.	Tolerant	[76]
Tinto Cão	Red	Portugal	Field Trial	Summer stress	Better adjustment of water status, minimized light-harvesting system, lower photosynthetic productivity, lower chlorophyll concentration, reduced photochemical efficiency, higher investment in photoprotective mechanisms.	Tolerant	[212]
Torrontés	White	Spain	Field trial	Summer stress	Higher stomatal density, length, and width.	Unconclusive	[192]
Touriga Franca	Red	Portugal	Field trial	Summer stress	Response varied between different locations. Sensitive to heat and drought and tolerant to high light in one location, while being tolerant to heat and light but sensitive to drought in another. High potential of adaptability.	Tolerant	[173]
Touriga Franca	Red	Portugal	Field trial	Drought (regulated deficit irrigation)	Lowest leaf temperature, highest stomatal conductance, low water use efficiency, lowest leaf area.	Tolerant (w/irrigation)	[107]
Touriga Nacional	Red	Portugal	Field trial	Summer stress	Smaller leaf size with lower dry weight and stomata density.	Less tolerant	[191]
Touriga Nacional	Red	Portugal	Growth chamber/field	Summer stress	High tolerance to heat and light stresses. No response to stress under controlled growth conditions. Fewer responsive genes under stress conditions.	Tolerant	[214]
Touriga Nacional	Red	Portugal	Growth chamber	Heat stress	Rapid and increased redox potential, increased photosynthetic pigments, increased ABA concentration, increased expression of heat-shock protein genes.	Tolerant	[97]
Touriga Nacional	Red	Portugal	Field trial	Summer stress	Moderate steam water potential, higher photosynthetic pigments concentration along with better photochemical responses. Gas exchange parameters remained stable throughout the analysis. Efficient use of radiation and CO_2_.	Tolerant	[212]
Touriga Nacional	Red	Portugal	Field trial	Summer stress	Highest soluble sugar content, highest water potential, highest net photosynthetic rate, highest stomatal conductance.	Tolerant	[76]
Treixadura	White	Spain	Field trial	Summer stress	Longer stomata. Stomatal density, length and width varied between years.	Unconclusive	[192]
Trincadeira	Red	Portugal	Field trial	Summer stress	Larger leaf size, higher leaf, and stomata density.	Less tolerant	[191]
Trincadeira	Red	Portugal	Growth chamber/field	Summer stress	Significant decreases in photosynthetic parameters. Higher number of responsive genes under stress, and a greater transcriptome reprogramming.	Sensitive	[214]
Trincadeira	Red	Portugal	Growth chamber	Heat stress	Slow and insufficient response to increased photosynthetic pigments, increased ABA concentration, increased expression of heat-shock protein and ROS scavenger genes.	Sensitive	[97]
Trincadeira	Red	Portugal	Field trial	Drought	Higher leaf temperature, lower stomatal conductance, gradual decrease of water potential, lower water use efficiency, lower net photosynthetic values.	Unconclusive	[107]
Trincadeira	Red	Portugal	Field trial	Drought (different irrigation regimes)	Drought plants recovered more rapidly and efficiently after irrigation. Drought regime led to decreased stomatal conductance and total chlorophyll. Reduced maximum assimilation rate, maximum and apparent quantum yield. Increased reflectance under drought. Increased Brix.	Tolerant	[201]
Valent Blanc	White	Spain	Field trial	Drought	Low stem water potential values.	Sensitive	[213]
Valent Negre	Red	Spain	Field trial	Drought	High intrinsic water use efficiency, low stem water potential values.	Sensitive	[213]
Veremeta	Red	Spain	Field trial	Summer stress	Higher stomatal conductance values.	Tolerant	[200]
Vinater Blanc	White	Spain	Field trial	Drought	Under progressive water depletion presented the high intrinsic water use efficiency.	Sensitive	[213]
Vinater Negre	Red	Spain	Field trial	Drought	Low stem water potential values, under progressive water depletion presented high intrinsic water use efficiency.	Tolerant	[213]
Viosinho	White	Portugal	Field trial	Summer stress	Smaller leaves with thicker upper epidermal cells and intermediate lower epidermal cells, thicker upper cuticle. Medium stomatal density.	Tolerant	[105]
Viosinho	White	Portugal	Field trial	Summer stress	Tolerant to heat, drought, and light stresses.	Tolerant	[173]

**Table 2:** Summary table of differentially expressed genes in grapevines under abiotic stress.

Gene	Protein	Organ	Type of Stress	Function/Hypothetical Function	Results	Reference
*CER1*	Fatty acyl-CoA reductase	Fruit	Drought	Aliphatic wax biosynthetic pathway	Upregulated	[210]
*CER2*	Fatty acyl-CoA reductase	Fruit	Drought	Aliphatic wax biosynthetic pathway	Upregulated	[210]
*CER3*	Fatty acyl-CoA reductase	Fruit	Drought	Aliphatic wax biosynthetic pathway	Upregulated	[210]
*CER4*	Fatty acyl-CoA reductase	Fruit	Drought	Aliphatic wax biosynthetic pathway	Upregulated	[210]
*CER10*	Fatty acyl-CoA reductase	Fruit	Drought	Aliphatic wax biosynthetic pathway	Upregulated	[210]
*Myb5a*	Transcription factor	Fruit	Drought	Affects the expression of several structural genes of the flavonoid pathway	Upregulated	[139]
*MybC*	Transcription factor/affects the expression of several structural genes of the flavonoid pathway	Fruit	Drought	Affects the expression of several structural genes of the flavonoid pathway	Upregulated	[139]
*VvCHS1*	Chalcone synthase	Fruit	Drought	Involved in flavonoid metabolism	No differences	[139]
*VvCHS2*	Chalcone synthase	Fruit	Drought	Involved in flavonoid metabolism	Upregulated	[139]
*VvCHS3*	Chalcone synthase	Fruit	Drought	Involved in flavonoid metabolism	Upregulated	[139]
*VvDFR*	Dihydroflavonol reductase	Fruit	Heat	Involved in flavonoid metabolism	Downregulated	[218]
*VvF3′5′Hi*	Flavonoid-3′5′-hydroxylase i	Fruit	Heat	Catalyse the hydroxylation of flavonoids	Downregulated	[218]
		Fruit	Drought		Upregulated	[139]
*VvF3′H*	Flavonoid 3′-hydroxylase	Fruit	Drought	Catalyse the hydroxylation of flavonoids	Upregulated	[139]
*VvF3′H A*	Flavonoid 3′-hydroxylase	Fruit	Drought	Phenylpropanoid pathway	No differences were observed	[232]
*VvF3′H B*	Flavonoid 3′-hydroxylase	Fruit	Drought	Phenylpropanoid pathway	Upregulated	[232]
*VvF3H*	Flavonoid 3-hydroxylase	Fruit	Drought	Catalyse the hydroxylation of flavonoids	Upregulated	[139]
*VvFLS*	Flavonol synthase	Fruit	Drought	Involved in flavonol biosynthesis	Upregulated in grafted grapevines	[232]
*VvGIN2*	Vacuolar invertase	Leaf	Drought	Involved in sugar transport	Upregulated	[221]
*VvGOLS1*	Galactinol synthase	Fruit	Heat	Biosynthesis of raffinose family oligosaccharides	Upregulated	[220]
*VvHsfA2*	Transcription factor	Fruit	Heat	Transcriptional factor of heat-stress related genes	Upregulated	[220]
		Leaf	Heat		Upregulated in the heat tolerant variety	[87]
*VvHSFA7*	Transcription factor	Leaf	Heat	Transcriptional factor of heat-stress related genes	Upregulated in the heat tolerant variety	[87]
*VvHSFA9*	Transcription factor	Leaf	Heat	Transcriptional factor of heat-stress related genes	Upregulated in the heat tolerant variety	[87]
*VvHT1*	Hexose transporter	Leaf	Drought	Involved in sugar transport	Downregulated	[221]
*VvHT5*	Hexose transporter	Leaf	Drought	Involved in sugar transport	Upregulated	[221]
*VvKCS12*	β-ketoacyl-CoA synthase	Leaf	Drought	Involved in cuticular wax biosynthesis	Upregulated	[231]
*VvKCS14*	β-ketoacyl-CoA synthase	Leaf	Drought	Involved in cuticular wax biosynthesis	Upregulated	[231]
*VvMSA*	ABA-, stress-, and ripening-induced protein	Leaf	Drought	Gene expression regulator under stress conditions	Upregulated	[221]
*VvMYB14*	Transcription factor	Fruit	Drought	Involved in secondary metabolism	Upregulated	[232]
*VvMYB4R1*	Transcription factor	Fruit	Drought	Transcriptional factor involved in stilbene biosynthesis	Upregulated	[232]
*VvMybA*	Transcription factor	Fruit	Heat	Regulate the expression of UFGT	Downregulated	[218]
		Fruit	Drought		Upregulated	[139]
*VvMYBC2-L3*	Transcription factor	Fruit	Drought	Transcriptional repressor in the synthesis of anthocyanins	Downregulated	[232]
*VvNAC44*	NAC domain-containing protein	Fruit	Drought	Involved in berry ripening and stress response	Upregulated	[232]
*VvNAC60*	NAC domain-containing protein	Fruit	Drought	Involved in berry ripening and stress response	Upregulated	[232]
*VvOMT*	O-methyltransferase	Fruit	Drought	Phenylpropanoid pathway	Upregulated	[139]
*VvPAL*	Phenylalanine ammonia-lyase	Fruit	Drought	Involved in the first step of the phenylpropanoid pathway	Upregulated	[232]
*VvPIP1;1*	Plasma membrane aquaporin	Leaf	Drought	Involved in the transport of water and small solutes	Differed between varieties	[229]
*VvPIP2;1*	Plasma membrane aquaporin	Leaf	Drought	Involved in the transport of water and small solutes	Downregulated	[229]
*VvPIP2;2*	Plasma membrane aquaporin	Leaf	Drought	Involved in the transport of water and small solutes	Differed between varieties	[229]
*VvPIP2;3*	Plasma membrane aquaporin	Leaf	Drought	Involved in the transport of water and small solutes	Differed between varieties	[229]
*VvPrx31*	Class III peroxidase	Fruit	Heat	Putative role in anthocyanin degradation	Upregulated	[218]
*VvPsbP*	Extrinsic subunit of photosystem II	Leaf	Heat	“Photosynthetic” pathway	Upregulated in the heat tolerant variety	[87]
*VvsHSP*	Small transcription factor	Leaf	Heat	Transcriptional factor of heat-stress related genes	Upregulated in the heat tolerant variety	[87]
*VvSUC11*	Sucrose transporter	Leaf	Drought	Involved in sugar transport	Upregulated	[221]
*VvTIP1;1*	Tonoplast aquaporins	Leaf	Drought	Involved in the transport of water and small solutes	Differed between varieties	[229]
*VvTIP2;1*	Tonoplast aquaporins	Leaf	Drought	Involved in the transport of water and small solutes	Downregulated	[229]
*VvUFGT*	UDP-glucose:flavonoid 3-O-glucosyltransferase	Fruit	Heat	Glycosylation of anthocyanidins	Downregulated	[218]
		Fruit	Drought		Upregulated	[139]
*WSD1*	Wax ester synthase/diacylglycerol acyltransferase 1	Fruit	Drought	Aliphatic wax biosynthetic pathway	Upregulated	[210]

## Data Availability

No new data were created or analysed in this study. Data sharing is not applicable to this article.
